# Effect of omega-3 supplementation on lipid profile in children and adolescents: a systematic review and meta-analysis of randomized clinical trials

**DOI:** 10.1186/s12937-022-00826-5

**Published:** 2023-02-10

**Authors:** Masoud Khorshidi, Zohreh Sajadi Hazaveh, Malek Alimohammadi-kamalabadi, Sanaz Jamshidi, Omid Moradi Moghaddam, Beheshteh Olang, Sayeh Hatefi, Amirhossein Hosseini, Parsa Jamilian, Meysam Zarezadeh, Parichehr Kohansal, Javad Heshmati, Parmida Jamilian, Aliakbar Sayyari

**Affiliations:** 1 Pediatric Gastroenterology, Hepatology and Nutrition Research Center, Research Institute for Children’s Health, Shahid Beheshti University of Medical Sciences, Tehran, Iran; 2 Department of Nutrition, School of Public Health, Iran University of Medical Sciences, Tehran, Iran; 3 Cancer Research Center, Shahid Beheshti University of Medical Sciences, Tehran, Iran; 4 Department of Clinical Nutrition, School of nutrition sciences and dietetics, Tehran University of Medical Sciences, Tehran, Iran; 5 Trauma and Injury Research Center, Critical Care Medicine Department, Iran University of Medical Sciences, Tehran, Iran; 6 Keele Medical School, Keele University, Staffordshire, UK; 7 Student Research Committee, Tabriz University of Medical Sciences, Tabriz, Iran; 8 Nutrition Research Center, School of Nutrition and Food Sciences, Tabriz University of Medical Sciences, Tabriz, Iran; 9 Department of Toxicology & Pharmacology, Faculty of Pharmacy, Tehran Medical Sciences, Islamic Azad University, Tehran, Iran; 10 Songhor Healthcare Center, Kermanshah University of Medical Sciences, Kermanshah, Iran; 11 School of Pharmacy and Bio engineering, Keele University, Staffordshire, UK; 12 Mofid Children Hospital, Tehran, 1546815514 Iran

**Keywords:** Omega-3, Lipid profile, Children, Systematic review, Meta-analysis

## Abstract

**Purpose:**

Dyslipidemia is considered as a known risk factor for cardiovascular disease. Yet various trials with wide ranges of doses and durations have reported contradictory results. We undertook this meta-analysis of randomized controlled trials (RCTs) to determine whether omega-3 supplementation can affect lipid profile in children and adolescents.

**Methods:**

Cochrane Library, Embase, PubMed, and Scopus databases were searched up to March 2021. Meta-analysis was performed using random-effect method. Effect size was expressed as weighted mean difference (WMD) and 95% confidence interval (CI). Heterogeneity was assessed using the I^**2**^ index. In order to identification of potential sources of heterogeneity, predefined subgroup and meta-regression analysis was conducted.

**Results:**

A total of 14 RCTs with 15 data sets were included. Based on the combination of effect sizes, there was a significant reduction in TG levels (WMD: -15.71 mg/dl, 95% CI: -25.76 to -5.65, *P*=0.002), with remarkable heterogeneity (I^2^=88.3%, *P*<0.001). However, subgroup analysis revealed that omega-3 supplementation significantly decreased TG only in studies conducted on participants ≤13 years old (WMD=-25.09, 95% CI: -43.29 to -6.90, *P*=0.007), (I^2^=84.6%, *P*<0.001) and those with hypertriglyceridemia (WMD=-28.26, 95% CI: -39.12 to -17.41, *P*<0.001), (I^2^=0.0%, *P*=0.934). Omega-3 supplementation had no significant effect on total cholesterol, HDL, and LDL levels. Also, results of nonlinear analysis showed significant effect of treatment duration on HDL status (P_non-linearity_=0.047).

**Conclusion:**

Omega-3 supplementation may significantly reduce TG levels in younger children and those with hypertriglyceridemia. Also, based on the HDL-related results, clinical trials with longer duration of intervention are recommended in this population.

## Introduction

Cardiovascular disease (CVD) is known as one of the major causes of death in the world, which is estimated to be on the rise [1]. Dyslipidemia is considered as a substantial risk factor for CVD [2]. It is characterized by increased levels of triglyceride and low density lipoprotein (LDL) and decreased levels of high density lipoprotein (HDL) [3, 4]. The prevalence of dyslipidemia in adolescents has been reported to be high [5], and increasing trend of obesity among children and adolescents can be considered as one of the possible causes for this matter [6]. So far, many drugs and food supplements such as omega-3 have been utilized to improve dyslipidemia in children.

Omega 3 fatty acids are a group of unsaturated fatty acids in which structurally the first double bond is located on third carbon in the carbon chain [7]. Since mammals' bodies are unable to produce these compounds, they must be included in the diet. Omega-3 unsaturated fatty acids (PUFA) are commonly found in vegetable oils and marine sources such as fish. Eicosapentaenoic acid (EPA) and docosahexaenoic acid (DHA) are the main fatty acids in fish oil [8]. Consumption of omega-3 supplements play an important role in reduction of CVD events and its associated mortality by ameliorating lipid profile via lowering triglyceride levels [9, 10]. Furthermore, accumulating evidence reported that total cholesterol, LDL-C and HDL-C levels are improved by n-3 fatty acids consumption through increasing fatty acid oxidation and reducing VLDL production [11, 12].

So far, many studies have been carried out concerning the effect of omega 3 on lipid profile in children and adolescents [13–15]. In most cases, the beneficial effects of omega 3 consumption in improvement of lipid profile have been noticed [16, 17]. However, some studies have not shown a significant effect of omega 3 on lipid profile [18, 19]. Various meta-analysis of omega-3 supplementation on lipid profile has been performed in adults [20, 21], but due to hormonal changes during puberty, it seems necessary to investigate this effect in children and adolescents. Hence, in this study, we aimed to evaluate the effect of omega 3 supplementation on lipid profile in children and adolescents.

## Methods

### Search strategy

The present study was conducted according to Muka et al. guideline and Cochrane handbook for systematic reviews of interventions [22]. PubMed (Medline), Cochrane Library, Scopus, and Embase databases and Google Scholar were searched by two reviewers independently up to March 2021. Following keywords and MeSH terms were used to search databases: ‘Hypercholesterolemia’ OR ‘HDL’ OR ‘LDL’ OR ‘Hyperlipidemias’ OR ‘Dyslipidemias’ OR ‘Triglycerides’ OR ‘Triacylglycerol’ OR ‘Lipid profile’ OR ‘Low density lipoprotein’ OR ‘High density lipoprotein’ OR ‘Cholesterol’ OR ‘Blood lipids’ AND ‘omega-3’ OR ‘Eicosapentaenoic acid’ OR ‘Docosahexaenoic acid’ OR ‘Fish oil’.

### Study selection

The current systematic review and meta-analysis include randomized clinical trials investigating the effect of omega-3 supplementation on lipid profile components as primary or secondary outcome. Human trials were included if they met the following inclusion criteria, including: 1) population: children and adolescents (age between 2 to 18 years old); 2) intervention: oral supplementation with omega-3; 3) study design: randomized clinical trials with either parallel or crossover design; 4) outcome: reporting mean ± standard deviation (or convertible equivalent) of lipid profile (HDL, LDL, TC and TG) at the baseline and the end of the study in each group. Studies were excluded if they: 1) were non-clinical trials; 2) had used omega-3 supplementation in combination with other agents; 3) reported insufficient information regarding lipid profile before and after the supplementation in placebo and intervention groups; 4) had not a placebo group. Two reviewers (S.H. and Z.S.) independently screened articles by title and abstract after removing duplicate manuscripts. Then, obtained articles went under assessment by full-text according to predefined inclusion criteria.

### Data extraction

Two reviewers independently screened and extracted study characteristics from the included studies including location, study population, first author, year, gender, sample size, duration of supplementation, omega-3 dose, and outcome data. Moreover, mean and standard deviation (SD) of the lipid profile components (HDL, LDL, TG, and TC) at the baseline and end of the intervention were extracted. For cross-over trials, only data from the first part of the study (before the washout period) was used for analysis. When the values for outcome variable were reported in different time points, data for the end of the trial were extracted. When the SD of the mean difference was not mentioned, it was calculated as follows: SD_change_= square root [(SD_baseline_^2^ + SD_final_^2^) - (2×R×SD_baseline_×SD_final_)]. Any discrepancies were resolved by discussion with a predetermined third reviewer (M.KH.).

### Quality assessment

Two independent investigators (Z.S., M.K.) assessed the risk of bias using the Cochrane collaboration's risk of bias assessment tool. For this, each study was assessed for seven criteria including (a) randomization generation, (b) allocation concealment, (c) blinding of participants and personnel, (d) blinding of outcomes assessors, (e) incomplete outcome data reporting, (f) selective reporting, (g) and the other sources of bias. Accordingly, studies were considered as high quality (low risk of bias for all seven domains), moderate quality (unclear risk of bias for one or two domains), and low quality (low risk of bias for less than two domains).

### Statistical analysis

In order to perform meta-analysis, random-effect model was employed. Also, between-study heterogeneity was identified by random-effect analysis. Heterogeneity was evaluated using the I^**2**^ index (I^2^ ≥50% and I^2^ <50% was considered as heterogeneous data and non-heterogeneous data, respectively) [23]. Stata 16.0 (Stata Corporation, College Station, TX) was employed to perform the statistical analysis of this study. Effect size was defined as weighted mean difference (WMD) and 95% confidence interval (CI). Standard deviation was calculated whenever the data were reported as standard error of the means (SEM) by multiplying SEM by the square root of the sample size. The effect sizes of meta-analysis were calculated based on mean differences and their corresponding standard deviations (SDs) of changes in lipid profile components for intervention and control groups [24]. Sensitivity analysis was performed in order to assess the influence of omitting each study on the overall effect size using the leave-one-out method. To identify potential sources of heterogeneity, predefined subgroup analysis was carried out based on suplementation period, mean age of participants, study population, and study design. In addition, we evaluated the presence of publication bias with the Egger's regression asymmetry tests and visual evaluation of the funnel plot [25]. Meta-regression analysis was done by a restricted maximum likelihood (REML) to evaluate the relation between the effect size duration of treatment as a potential moderator variable (omega-3 dose was not used in regression analysis and subgroup analysis because some studies provided only one pure type of omega-3 fatty acids, for example DHA, and some studies performed supplementation in terms of participants' body weight). This method corresponds to random-effects meta-regression comprising both within-study variances of treatment effects and the residual between-study heterogeneity. We also executed fractional polynominal modeling (polynomials) to explore the non-linear potential effects of omega-3 duration of treatment (months). Covariates for the meta-regression analysis were defined based on evidence-based knowledge. *P* < 0.05 was considered as significance level.

## Results

### Study selection and data obtaining

**Fig. 1:**
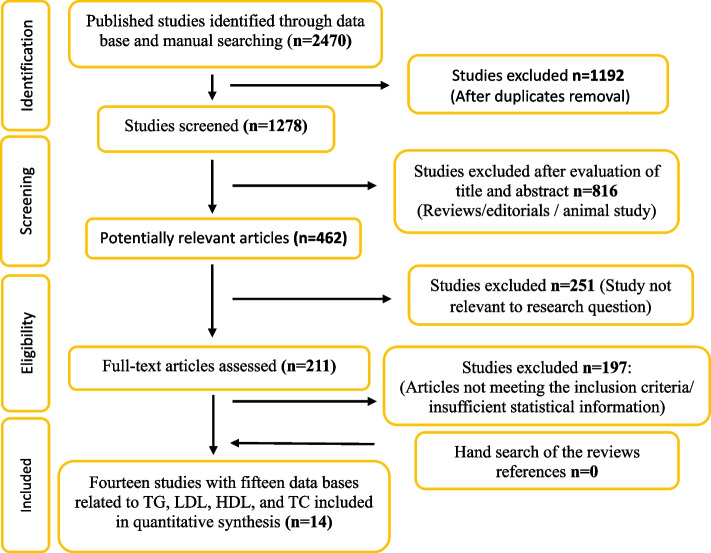
Flow diagram of the review progress

From the primary searches, 2470 eligible records were identified, which is illustrated in Fig. 1. After excluding duplications, 1278 studies remained for title and abstract evaluation. Full texts of 211 records was read and 14 articles met our inclusion criteria. Out of 14 studies, 14, 12, 12 and 8 studies reported the effect of omega 3 on TG, TC, HDL, and LDL, respectively.

### Study and participant characteristics

Table 1 revealed the characteristics of the 14 studies and their contributing data to our meta-analysis. Three trials out of 14 had cross-over design, while others were parallel randomized trials. Studies were conducted between 2001 to 2019 in Italy, Iran, Spain, Egypt, Turkey, USA, Mexico, Poland, and Denmark. Enrolled participants were from both sexes except for one study which was conducted on male subjects. Totally, 848 participants were enrolled in our meta-analysis and sample sizes varied from 20 to 130 participants and 10 to 15 years of age. Studies was carried out in patients with hyperphenylalaninemia, metabolic syndrome, methylmalonic academia, hemodialysis, NAFLD, hypertriglyceridemia, obesity, and migraine. Also, the duration of studies was varied between 2 and 24 months.

**Table 1:** Characteristics of studies included in the meta-analysis

Author and year	Country	Study population	Sex	Dose (mg/d)	Duration (month)	Sample size	Age	Study design	Outcome
Agostoni et al. (2001)	Italy	Hyperphenylalaninemia	F/M	one 500 mg capsule LCPUFA/4 Kg body weight	12	20	10	parallel	LDL, TG, HDL, TC.
Ahmadi et al. (2014)	Iran	Metabolic Syndrome	F/M	omega-3 tablets(2.4 gr/day)	2	53	14	parallel	LDL, TG, HDL, TC.
Ald´ amiz et al. (2006)	Spain	Methylmalonic acidemia	F/M	25 mg/kg per day DHA	3	60	10	crossover	TG, HDL, TC.
Ateya et al. (2017) [17]	Egypt	Hemodialysis	F/M	1-g oral omega-3 capsule	4	49	14.7	parallel	LDL, TG, HDL, TC.
Boyraz et al. (2015) [26]	Turkey	NAFLD	F/M	1000 mg dose of PUFA	12	108	13.8	parallel	LDL, TG, HDL, TC.
de Ferranti et al. (2014) [14]	USA	Hypertriglyceridemia	F/M	1 g capsule ( 840 mg omega-3 FA, 465 mg EPA and 375 mg DHA, in 4 mg of carrier vegetable oil)	6	21	14.5	parallel	LDL, TG, HDL, TC.
Del-Río-Navarro et al. (2019) [16]	Mexico	Obese	F/M	3 g/day of omega-3	3	130	13	parallel	TG, HDL, TC.
Gidding et al. (2014) [15]	USA	Hypertriglyceridemia	F/M	4 g of fish oil	2	42	14	crossover	LDL, TG, HDL, TC.
Harel et al. (2002)	USA	Migraine	F/M	2 capsules (1-g n-3/day each)	2	27	15	crossover	TG.
Huang et al. (2019)	Mexico	Hypertriglyceridemia	F/M	3 g/day of 3 PUFAs supplementation	3	65	12.6	parallel	TG, HDL, TC.
Janczyk et al. (2015)	Poland	NAFLD	F/M	450-1300 w3 mg/day	6	64	13.1	parallel	LDL, TG, HDL, TC.
Nobili et al. (250mg) (2013)	Italy	NAFLD	F/M	250 mg DHA/d	24	40	12	parallel	TG.
Nobili et al. (500mg) (2013)	Italy	NAFLD	F/M	500 mg DHA/d	24	40	12	parallel	TG.
Pacifico (2015)	Italy	NAFLD	F/M	250 mg DHA/day	6	51	10.9	parallel	TG, HDL, TC.
Pedersen (2010)	Denmark	Metabolic Syndrome	M	1.5 g of n-3/d	4	78	14	parallel	LDL, TG, HDL, TC.

*NAFLD* nonalcoholic fatty liver disease ovary syndrome, *TC* total cholesterol, *TG* triglyceride, *HDL* high density lipoprotein, *LDL* low density lipoprotein, *LCPUFA* long chain poly unsaturated fatty acid

### Quality of included studies and risk of bias

**Fig. 2:**
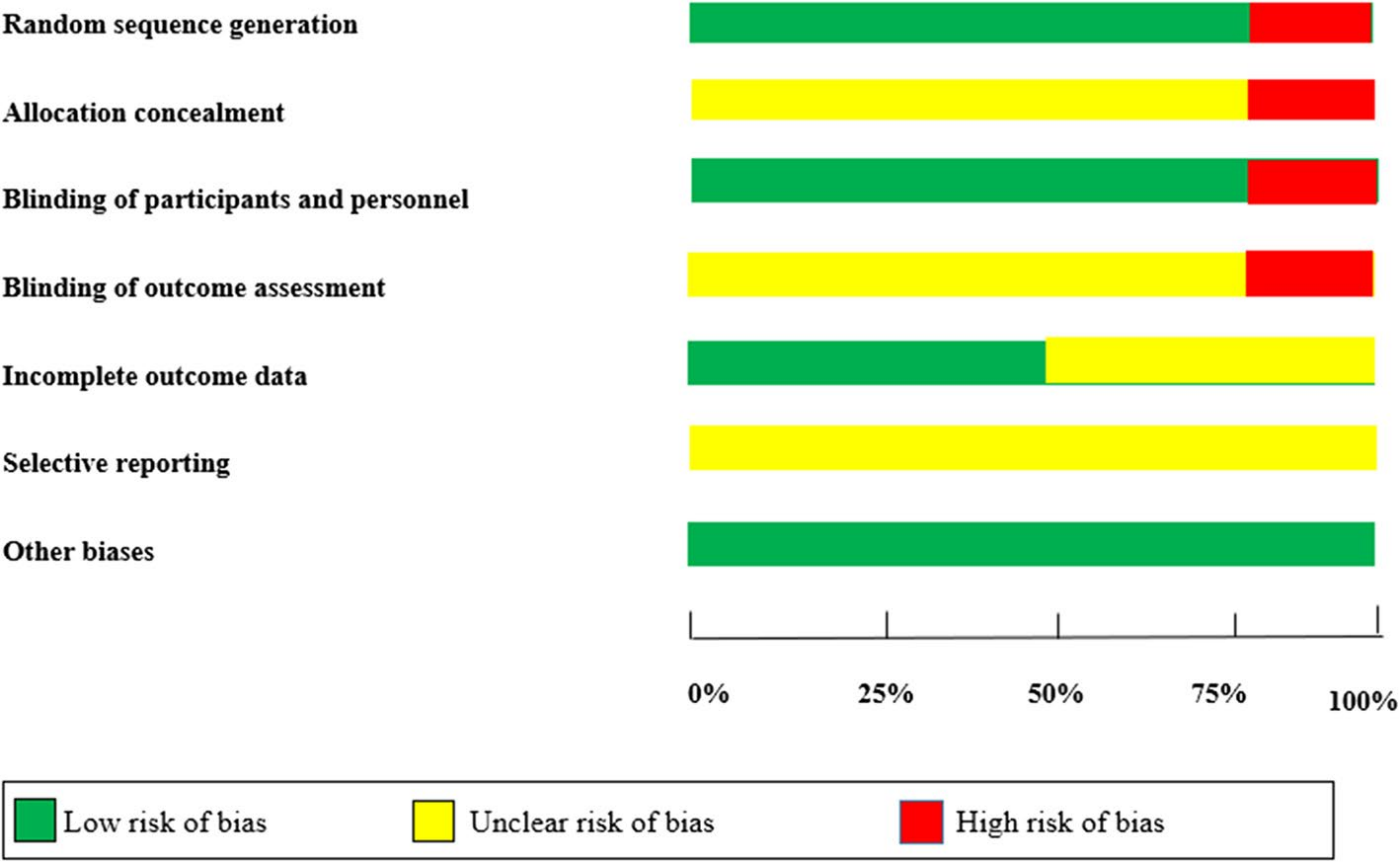
Assessment of quality of studies by the Cochrane Collaboration's tool

Summary assessments of the risk of bias is presented in Fig. 2 and Table 2. Random sequence generation was judged to be low in all trials except for one. Evaluation of allocation concealment revealed 10 studies with unclear risk of bias. Blinding of participants and blinding of outcomes assessment showed 9 and 6 studies with low risk of bias. All studies had low risk of incomplete outcome data and selective reporting. Also, risk of other biases was unclear in eleven studies.

### Effect of omega-3 consumption on TG concentrations

**Fig. 3:**
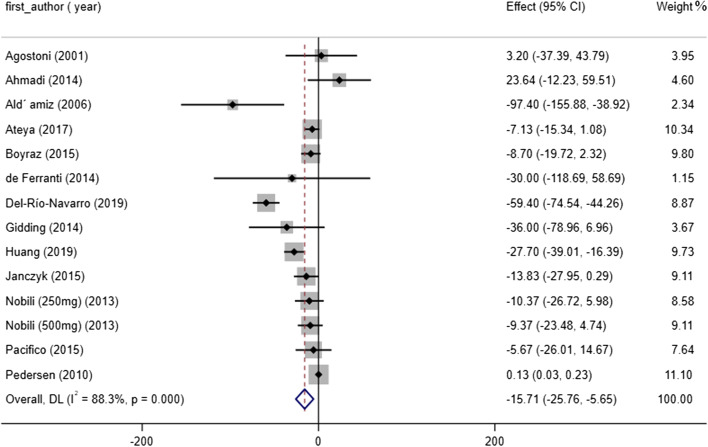
Forest plot detailing WMD and 95% CIs for the effect of omega-3 supplementation on TG

**Fig. 4:**
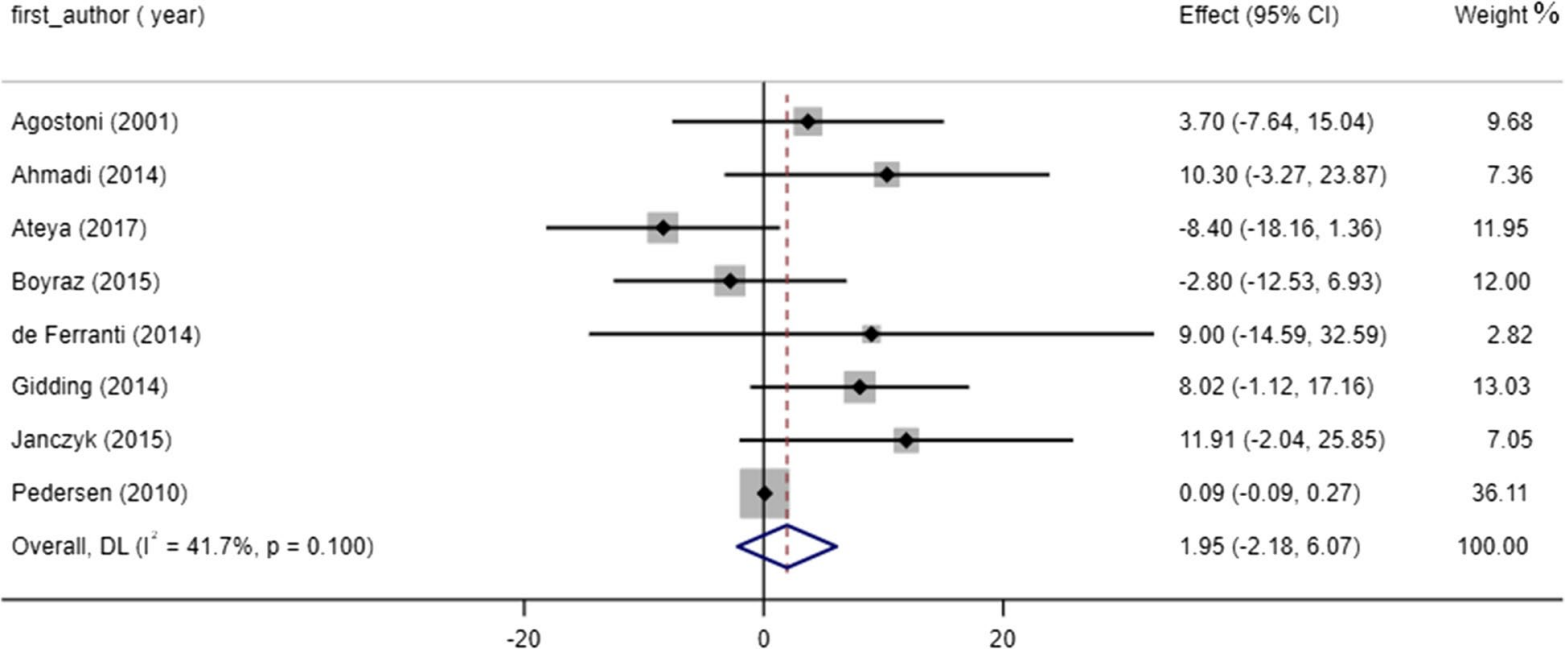
Forest plot detailing WMD and 95% CIs for the effect of omega-3 supplementation on LDL

Determination of whether omega-3 supplementation affects TG status indicated a significant reduction in TG levels (WMD: -15.71 mg/dl, 95% CI: -25.76 to -5.65, *P* = 0.002), and also significant heterogeneity was reported (I^2^ = 88.3%, *P* < 0.001) (Fig. 3). Subgroup analysis based on age, study design, duration and population revealed that omega-3 supplementation had significant effect on TG reduction in studies conducted on participants ≤13 years old (WMD = -25.09, 95% CI: -43.29 to -6.90, *P* = 0.007), (I^2^ = 84.6%, *P* < 0.001) and those with hypertriglyceridemia (WMD = -28.26, 95% CI: -39.12 to -17.41, *P* < 0.001), (I^2^ = 0.0%, *P* = 0.934) (Table 3). Based on meta-regression analysis, pooled estimate is independent of study duration (slope: 0.693; 95% CI: -1.171 to 2.558; *P* = 0.434). Moreover, we failed to find a significant effect of treatment duration on TG levels based on non-linear meta-analysis (P _non-linearity_ = 0.226). Step by step exclusion of a single or few trials from analysis indicates independency of pooled effect size from each study Fig 4.

**Table 2:** Risk of bias assessment according to the Cochrane collaboration’s risk of bias assessment tool

Study, Year (reference)	Random sequence generation	Allocation concealment	Blinding of participants and personnel	Blinding of outcome assessment	Incomplete outcome data	Selective reporting	Other biases	Overall assessment
Agostoni et al., 2001	Low	Unclear	Low	Unclear	Low	Low	Unclear	Moderate quality
Ahmadi et al., 2014	Low	Unclear	Low	Unclear	Low	Low	Unclear	Moderate quality
Ald’ amiz et al., 2006	Unclear	Unclear	High	High	Low	Low	Unclear	Moderate quality
Ateya et al., 2017 [17]	Low	Unclear	Low	Unclear	Low	Low	Unclear	Moderate quality
Boyraz et al., 2015 [26]	Low	Unclear	Unclear	Low	Low	Low	Unclear	Moderate quality
de Ferranti et al., 2014 [14]	Low	Low	Low	Low	Low	Low	Low	High quality
Del-Río-Navarro et al., 2019 [16]	Low	Unclear	Low	Low	Low	Low	Unclear	Moderate quality
Gidding et al., 2014 [15]	Low	Unclear	Low	Unclear l	Low	Low	Unclear	Moderate quality
Harel et al., 2002	Low	Unclear	Unclear	Unclear	Low	Low	Low	Moderate quality
Huang et al., 2019	Low	Unclear	Unclear	Unclear	Low	Low	Unclear	Moderate quality
Janczyk et al., 2015	Low	Low	Unclear	Unclear	Low	Low	Unclear	Moderate quality
Nobili et al., 2013	Low	Low	Low	Low	Low	Low	Unclear	Moderate quality
Pacifico et al., 2015	Low	Low	Low	Low	Low	Low	Low	High quality
Pedersen et al., 2010	Low	Unclear	Low	Low	Low	Low	Unclear	Moderate quality

**Table 3:** Subgroup analysis of included RCTs in meta-analysis

Variable	Age		Study design		Trial duration		Study Population		
**TG**	**> 13 years**	**≤ 13 years**	Parallel	Cross over	**≥ 6 months**	**< 6months**	**E**	**N**	**H**
No. of comparison	7	7	12	2	7	7	3	8	3
WMD (95% CI)	-5.59(-12.58, 1.40)	-25.09(-43.29, -6.90)	-12.95(-22.88, -3.01)	-63.36(-123.18, -3.55)	-9.63(-15.94, -3.33)	-23.41(-41.37, -5.46)	-25.98(-69.35, 17.40)	-12.18(-25.19, 0.82)	-28.26(-39.12, -17.41)
p value	0.117	0.007	0.011	0.038	0.003	0.011	0.240	0.066	0.000
I^2^ (%)	57.2	84.6	88.7	63.6	0.0	94	78.5	90.1	0.0
p-heterogeneity	0.029	0.000	0.000	0.097	0.981	0.000	0.010	0.000	0.934
**HDL**	**> 13 years**	**≤ 13 years**	Parallel	Cross over	**≥ 6 months**	**< 6months**	**E**	**N**	**H**
No. of comparison	7	5	10	2	5	7	3	6	3
WMD (95% CI)	1.89 (-0.61, 4.38)	-0.06 (-3.82, 3.70)	0.92 (-1.02, 2.86)	5.72 (-8.40, 19.84)	3.13(0.20, 6.07)	-0.71(-2.67, 1.24)	4.62 (-6.40, 16.84)	-2.02(-2.97, 0.71)	-1.04 (-3.74, 1.66)
p value	0.138	0.974	0.352	0.428	0.036	0.473	0.211	0.443	0.451
I^2^ (%)	88.7	78.3	88.7	52.7	75.6	75.4	64.6	82.3	0.0
p-heterogeneity	0.000	0.001	0.000	0.146	0.003	0.000	0.059	0.000	0.926
**LDL**	**> 13 years**	**≤ 13 years**	Parallel	Cross over	**≥ 6 months**	**< 6months**	**E**	**N**	**H**
No. of comparison	7	1	7	1	4	4	2	4	2
WMD (95% CI)	1.94 (-2.75, 6.64)	3.70(-7.64, 15.04)	0.92 (-3.24, 5.07)	8.02 (-1.12, 17.16)	3.18(-3.39, 9.74)	1.52 (-4.67, 7.71)	-2.71(-14.55, 9.13)	2.21(-3.25, 7.67)	8.15 (-0.37, 16.67)
p value	0.417	0.523	0.665	0.085	0.343	0.631	0.654	0.428	0.061
I^2^ (%)	48.3	0.0	34.2	0.0	6.1	62.4	60.2	43.1	0.0
p-heterogeneity	0.071	-	0.167	-	0.363	0.047	0.113	0.153	0.939
**TC**	**> 13 years**	**≤ 13 years**	Parallel	Cross over	**≥ 6 months**	**< 6months**	**E**	**N**	**H**
No. of comparison	7	5	10	2	5	7	3	6	3
WMD (95% CI)	-2.09(-14.67, 10.49)	-0.68(-9.83, 8.48)	-1.64(-10.21, 6.94)	0.55(-10.83, 11.92)	3.84(-1.98, 9.67)	-5.43(-17.04, 6.19)	-11.62 (-48.48, 25.24)	1.99 (-4.78, 8.77)	-0.76(-7.60, 6.09)
p value	0.745	0.885	0.708	0.925	0.196	0.360	0.537	0.564	0.828
I^2^ (%)	93.3	65.4	91.4	0.0	0.0	94.1	94.3	78.2	0.0
p-heterogeneity	0.000	0.021	0.000	0.797	0.725	0.000	0.000	0.000	0.796

*TC* total cholesterol, *TG* triglyceride, *HDL* high density lipoprotein, *LDL* low density lipoprotein, *H* hyperlipidemia, N: includes patients with NAFLD, obesity and metabolic syndrome; E: Includes patients with migraine or hemodialysis or hyperphenyl alaninemia or methylmalonic acidemia

### Effect of omega-3 consumption on HDL concentrations

**Fig. 5:**
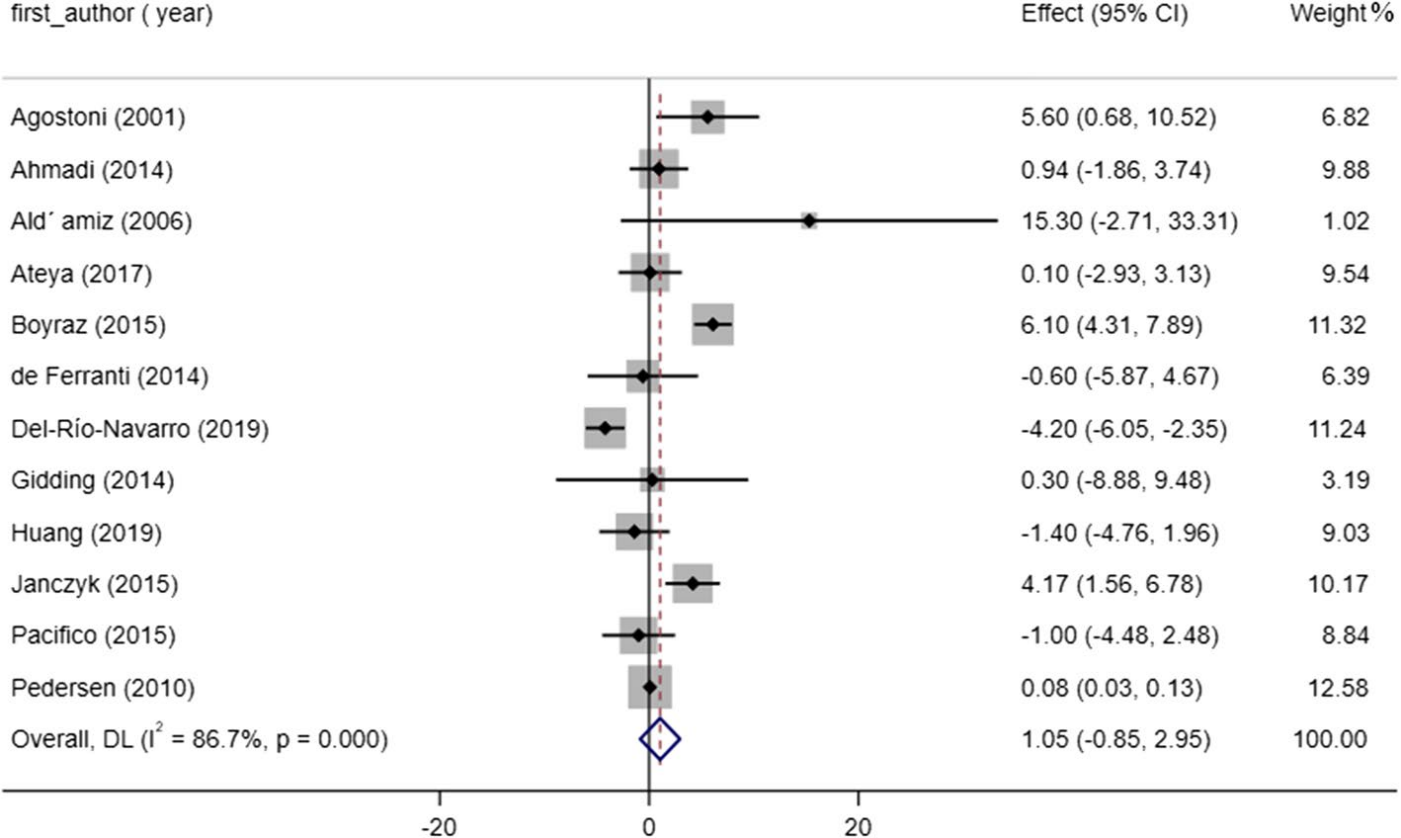
Forest plot detailing WMD and 95% CIs for the effect of omega-3 supplementation on HDL

We examined the possible influence of omega-3 supplementation on HDL by extracting data from the eligible studies. The effect of omega-3 on HDL was reported insignificant (WMD: 1.05, 95% CI: -0.85 to 2.95, P = 0.27). There was also substantial evidence of heterogeneity between studies (I^2^ = 86.7%, *P* < 0.001) (Fig. 5). In addition, results of sub-group analysis showed no significant effect of omega-3 treatment on HDL status (Table 3). A subsequent meta-regression analysis revealed a significant difference in HDL levels based on study duration (slope: 0.745; 95% CI: 0.269 to 1.221; *p* = 0.006). Also, results from the nonlinear analysis showed significant effect of treatment duration on HDL status (P _non-linearity_ = 0.047). Sensitivity analysis indicates that the pooled effect size did not depend on any of studies.

### Effect of omega-3 consumption on LDL concentrations

Pooling effect sizes of 8 studies reported no significant effect of omega-3 treatment on LDL status (WMD: 1.95, 95% CI: -2.18 to 6.07, P = 0.35). In addition, no significant heterogeneity was detected between studies (I^2^ = 41.7%, P = 0.1) (Fig. 4). Also, no significant effect of omega-3 supplementation was seen on LDL level based on sub-group analysis (Table 3). A meta-regression analysis was performed based on the duration of omega-3 intervention subgroups and demonstrated no significant differences (slope: -0.370; 95% CI: -2.044 to 1.303; *p* = 0.608). With regards to the non-linear analysis, no remarkable association was seen between study duration and LDL level (P _non-linearity_ = 0.137). In addition, the pooled effect size was independent from a single or few studies.

### Effect of omega-3 consumption on TC concentrations

**Fig. 6:**
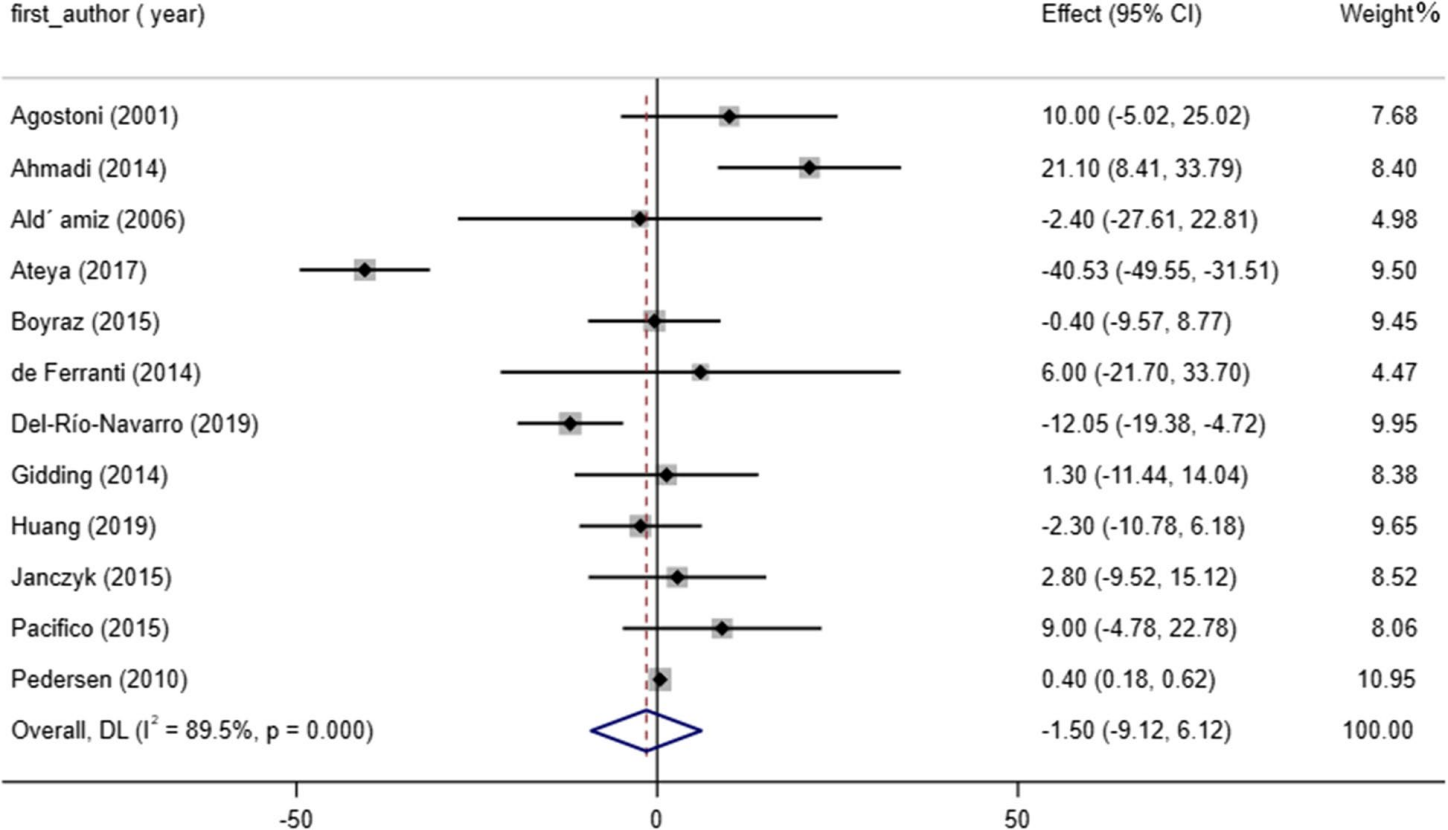
Forest plot detailing WMD and 95% CIs for the effect of omega-3 supplementation on TC

**Fig. 7:**
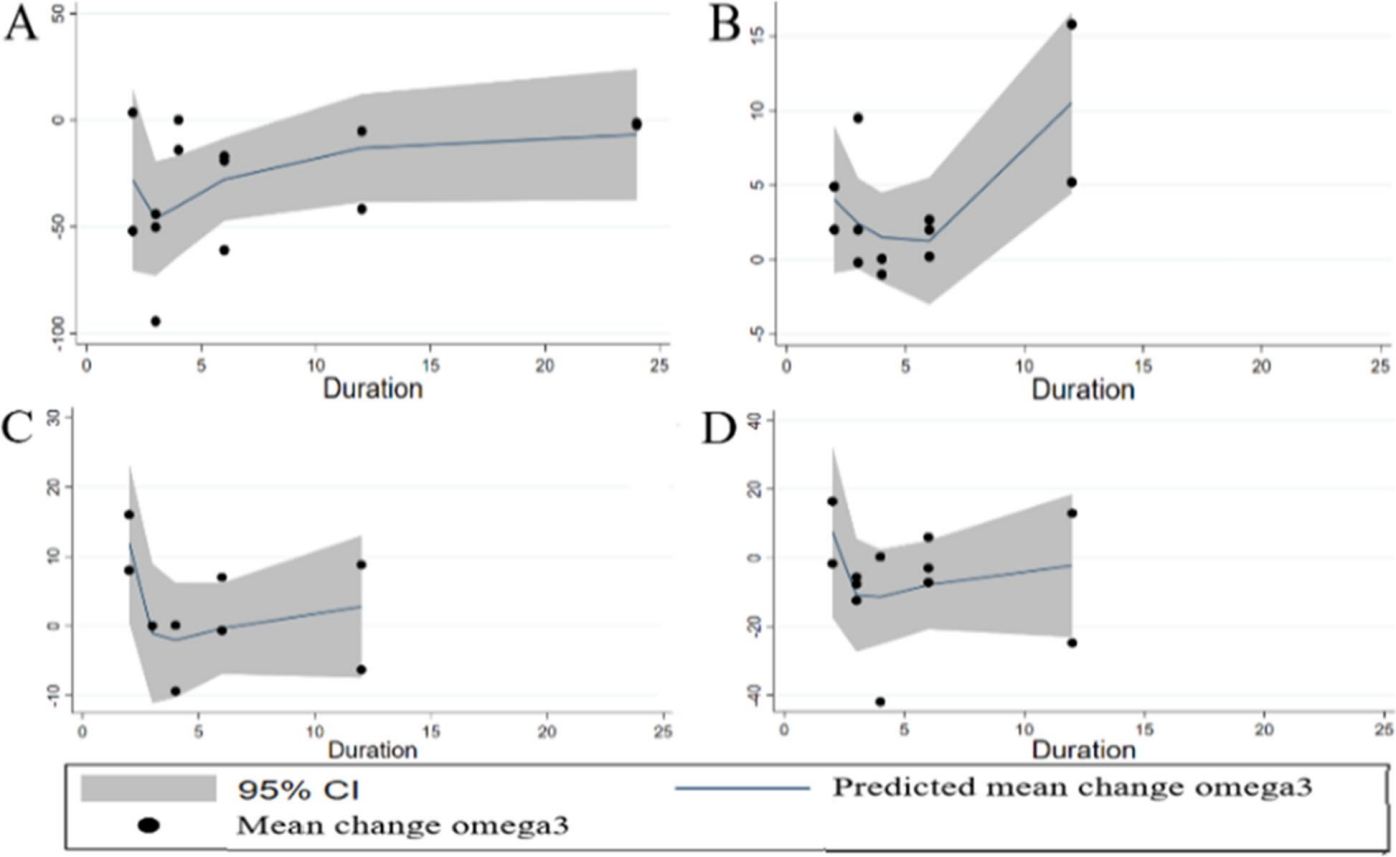
Dose-response relations between omega-3 duration of treatment (mg/d) and mean difference in TG, LDL, HDL, and TC

Meta-analysis of 12 studies showed no significant effect of omega-3 consumption on TC levels (WMD: -1.50, 95% CI: -9.12 to 6.12, *P* = 0.69). There was an apparent heterogeneity between trials (I^2^ = 89.5%, *P* < 0.001) (Fig. 6). Performed subgroup analysis indicated no remarkable difference in TC status due to omega-3 intake (Table 3). Our meta-regression analysis revealed no remarkable difference in TC level with respect to the study duration (slope: 0.699; 95% CI: -2.483 to 3.882; *P* = 0.635). Also, study duration didn’t influence TC due to the non-linear analysis (P non-linearity = 0.381). Due to sensitivity analysis, no single study likely affected the pooled results Fig 7.

### Publication bias

According to the funnel plot, no evidence of publication bias was seen for HDL, LDL and TC. However, there was a clue of publication bias for TG. Similarly, Egger’s test approved these findings and reports remarkable risk of bias for TG (*p* = 0.01), but not for HDL, LDL, and TC (*P* = 0.41, *p* = 0.29, and *p* = 0.55, respectively).

## Discussion

Herein, we found that omega-3 supplementation may exert therapeutic effects on TG levels. However, no remarkable effect of omega-3 intake was seen on HDL, LDL, and TC status. Also, subgroup analysis revealed an improvement in TG levels in studies conducted on participants ≤13 years old and those with hypertriglyceridemia. In addition, due to meta-regression results, the omega-3 supplementation improved HDL levels when administered with longer duration.

Omega-3 significantly decreased TG levels in our meta-analysis and specially in those younger than 13 and experiencing hypertriglyceridemia. In line with our findings, a systematic review and meta-analysis by Natto et al. revealed that omega-3 administration cause a significant TG reduction (−44.88 mg/dL 95% CI: −82.6, −7.16, *p* < 0.0001) in diabetic patients. However, no significant change was observed in patients with cardiovascular disorders [27]. Similarly, in the study performed by Chauhan et al. , administration of omega-3 was effective in reducing TG levels in diabetic dyslipidemia [28]. Although, another meta-analysis and meta-regression of RCTs indicated no significant association between omega-3 PUFA intake and all lipid markers in type 2 diabetes [20].

The exact mechanism by which omega-3 PUFAs had TG lowering effects returns to high affinity of omega-3 for peroxisome proliferator activated receptor (PPAR) followed by enhancement of beta oxidation and fatty acid metabolism. Moreover, omega-3 may decrease hepatic TG synthesis through inhibition of acyl coA1, 2 diacylglycerol acyl transferase [28, 29]. Also, omega-3 fatty acids stimulate other nuclear receptors including hepatocyte nuclear factor 4α, liver X receptor and farnesol X receptor, which modulates TG levels [30].

We failed to find significant effect of omega-3 on HDL, LDL, and TC levels in the current study, which is consistent with previously published research projects. A meta-Analysis by Chen et al. revealed that omega-3 supplementation in patients suffered from type 2 diabetes resulted in a reduction in TG concentrations, while no marked effect was observed on TC levels [21]. Also, a double-blind, randomized, multicenter trial with 1384 participants and 4 grams per day omega-3 supplementation didn’t show beneficial effect on lipid profile [31]. In addition, Hasan et al. indicated that omega-3 administration in peritoneal dialysis patients decreased TG levels significantly, with no effect on TC, HDL, and LDL status [32]. However, there might be some factors influencing our results. Using different types of omega-3 (PUFA, DHA, EPA, or mixture of all) supplements in included studies may affect the overall results. Moreover, using various types of oil (olive, sunflower, corn, and etc.) in control groups may be a justification for not observing meaningful results.

Although the effect of omega-3 consumption was not significant in our meta-analysis of total data, meta-regression results indicated an improvement in HDL levels based on intervention period. Our findings were in agreement with a study by Boyraz et al., which was conducted on adolescents with NAFLD and showed an improvement in HDL and TG levels after 12 months of 1gr/day PUFA intervention [26]. Although the exact mechanism through which omega-3 might influence HDL have not been still understood, some evidence detected that HDL often is likely to increase when there was a marked reduction in serum TG concentrations [33]. PUFA inhibits SREBP-1c and lipogenesis, while increases PPAR-α and fatty acid oxidation [34]. Through these mechanisms, PPAR-α agonists might be able to lower TG and increase HDL levels [35].

To the best of our knowledge, our study is the first systematic review and meta-analysis conducted to find the effect of omega-3 supplementation on lipid profile in children and adolescents. However, it has few limitations. First, the percentage of heterogeneity was high for almost all parameters. Second, studies have been conducted on different types of diseases. It should be mentioned that more reliable results may obtain when acceptable number of included studies were conducted on the patients with same disease. And third, there was the possibility of publication bias for included studies with TG level report.

## Conclusion

Based on the available evidence, omega-3 supplementation may have favorable hypolipidemic effects through reduction of TG levels. Since these improvements were observed in younger children and those with hypertriglyceridemia, clinicians should be aware of these beneficial effects. Also, based on HDL-related results, clinical trials with longer duration of intervention and appropriate designs are recommended in this population.

## Data Availability

not applicable.

## References

[1] Laslett LJ, Alagona P, Clark BA, Drozda JP, Saldivar F, Wilson SR (2012). The worldwide environment of cardiovascular disease: prevalence, diagnosis, therapy, and policy issues: a report from the American College of Cardiology. J Am Coll Cardiol.

[2] Nelson RH (2013). Hyperlipidemia as a risk factor for cardiovascular disease. Prim Care.

[3] Franssen R, Monajemi H, Stroes ES, Kastelein JJ (2011). Obesity and dyslipidemia. Med Clin North Am.

[4] Gau GT, Wright RS (2006). Pathophysiology, diagnosis, and management of dyslipidemia. Curr Probl Cardiol.

[5] Tomeleri CM, Ronque ER, Silva DR, Júnior CGC, Fernandes RA, Teixeira DC (2015). Prevalence of dyslipidemia in adolescents: comparison between definitions. Rev Port Cardiol.

[6] Cali AM, Caprio S (2008). Obesity in children and adolescents. J Clin Endocrinol Metabol.

[7] Calder PC, Yaqoob P (2009). Understanding omega-3 polyunsaturated fatty acids. Postgrad Med.

[8] Whelan J, Rust C (2006). Innovative dietary sources of n-3 fatty acids. Annu Rev Nutr.

[9] Ibrahim A, Hasan AA, Adel H, Yousif E (2014). Investigate the effect of omega-3 on lipid profile. Eur J Mol Bio Biochem.

[10] Gazi I, Liberopoulos EN, Saougos VG, Elisaf M (2006). Beneficial effects of omega-3 fatty acids: the current evidence. Hellenic J Cardiol.

[11] Schiano V, Laurenzano E, Brevetti G, De Maio JI, Lanero S, Scopacasa F (2008). Omega-3 polyunsaturated fatty acid in peripheral arterial disease: effect on lipid pattern, disease severity, inflammation profile, and endothelial function. Clin Nutr.

[12] Ashcheulova Т, Gerasimchuk N, Demydenko G, Kompaniiets K, Kochubiei O (2017). Dislipidemia: definition, diagnostics and treatment. Lik Sprava.

[13] Damsgaard CT, Schack-Nielsen L, Michaelsen KF, Fruekilde M-B, Hels O, Lauritzen L (2006). Fish oil affects blood pressure and the plasma lipid profile in healthy Danish infants. J Nutr.

[14] de Ferranti SD, Milliren CE, Denhoff ER, Steltz SK, Selamet Tierney ES, Feldman HA (2014). Using high-dose omega-3 fatty acid supplements to lower triglyceride levels in 10-to 19-year-olds. Clin Pediatr.

[15] Gidding SS, Prospero C, Hossain J, Zappalla F, Balagopal PB, Falkner B (2014). A double-blind randomized trial of fish oil to lower triglycerides and improve cardiometabolic risk in adolescents. J Pediatr.

[16] Del-Río-Navarro BE, Miranda-Lora AL, Huang F, Hall-Mondragon MS, Leija-Martínez JJ (2019). Effect of supplementation with omega-3 fatty acids on hypertriglyceridemia in pediatric patients with obesity. J Pediatr Endocrinol Metab.

[17] Ateya AM, Sabri NA, El Hakim I, Shaheen SM (2017). Effect of omega-3 fatty acids on serum lipid profile and oxidative stress in pediatric patients on regular hemodialysis: A randomized placebo-controlled study. J Ren Nutr.

[18] Chahal N, Manlhiot C, Wong H, McCrindle BW (2014). Effectiveness of omega-3 polysaturated fatty acids (fish oil) supplementation for treating hypertriglyceridemia in children and adolescents. Clin Pediatr.

[19] Del Bo C, Deon V, Abello F, Massini G, Porrini M, Riso P (2019). Eight-week hempseed oil intervention improves the fatty acid composition of erythrocyte phospholipids and the omega-3 index, but does not affect the lipid profile in children and adolescents with primary hyperlipidemia. Food Res Int.

[20] O’Mahoney LL, Matu J, Price OJ, Birch KM, Ajjan RA, Farrar D (2018). Omega-3 polyunsaturated fatty acids favourably modulate cardiometabolic biomarkers in type 2 diabetes: a meta-analysis and meta-regression of randomized controlled trials. Cardiovasc Diabetol.

[21] Chen C, Yu X, Shao S (2015). Effects of omega-3 fatty acid supplementation on glucose control and lipid levels in type 2 diabetes: a meta-analysis. PLoS One.

[22] Muka T, Glisic M, Milic J, Verhoog S, Bohlius J, Bramer W (2020). A 24-step guide on how to design, conduct, and successfully publish a systematic review and meta-analysis in medical research. Eur J Epidemiol.

[23] Higgins J, Green S (2011). Cochrane Handbook for Systematic Reviews of Interventions, 9.4. 6 Combining Dichotomous and Continuous Outcomes. Cochrane Handbook for Systematic Reviews of Interventions.

[24] Wu Y, Zhang Q, Ren Y, Ruan Z (2017). Effect of probiotic Lactobacillus on lipid profile: A systematic review and meta-analysis of randomized, controlled trials. PLoS One.

[25] Egger M, Smith GD, Schneider M, Minder C (1997). Bias in meta-analysis detected by a simple, graphical test. Bmj..

[26] Boyraz M, Pirgon Ö, Dündar B, Çekmez F, Hatipoğlu N (2015). Long-term treatment with n-3 polyunsaturated fatty acids as a monotherapy in children with nonalcoholic fatty liver disease. J Clin Res Pediatr Endocrinol.

[27] Natto ZS, Yaghmoor W, Alshaeri HK, Van Dyke TE (2019). Omega-3 fatty acids effects on inflammatory biomarkers and lipid profiles among diabetic and cardiovascular disease patients: a systematic review and meta-analysis. Sci Rep.

[28] Chauhan S, Kodali H, Noor J, Ramteke K, Gawai V (2017). Role of omega-3 fatty acids on lipid profile in diabetic dyslipidaemia: single blind, randomised clinical trial. J Clin Diagn Res.

[29] Hoy SM, Keating GM (2009). Omega-3 ethylester concentrate. Drugs..

[30] Davidson MH (2006). Mechanisms for the hypotriglyceridemic effect of marine omega-3 fatty acids. Am J Cardiol.

[31] Nicholls SJ, Lincoff AM, Garcia M, Bash D, Ballantyne CM, Barter PJ (2020). Effect of high-dose omega-3 fatty acids vs corn oil on major adverse cardiovascular events in patients at high cardiovascular risk: the STRENGTH randomized clinical trial. Jama..

[32] Hassan KS, Hassan SK, Hijazi EG, Khazim KO (2010). Effects of omega-3 on lipid profile and inflammation markers in peritoneal dialysis patients. Ren Fail.

[33] Skulas-Ray AC, West SG, Davidson MH, Kris-Etherton PM (2008). Omega-3 fatty acid concentrates in the treatment of moderate hypertriglyceridemia. Expert Opin Pharmacother.

[34] Spadaro L, Magliocco O, Spampinato D, Piro S, Oliveri C, Alagona C (2008). Effects of n-3 polyunsaturated fatty acids in subjects with nonalcoholic fatty liver disease. Dig Liver Dis.

[35] Balachandran K (2019). Dual PPAR α/γ agonists: Continuing cardiac concerns. Indian J Endocrinol Metabol.

